# Predictors of Effects of Lifestyle Intervention on Diabetes Mellitus Type 2 Patients

**DOI:** 10.1100/2012/962951

**Published:** 2012-04-19

**Authors:** Ramune Jacobsen, Eva Vadstrup, Michael Røder, Anne Frølich

**Affiliations:** ^1^Section for Social Pharmacy, University of Copenhagen, Jagtvej 160, 1st Floor, 2400 Copenhagen, Denmark; ^2^Department of Oncology, Rigshospitalet, Blegdamsvej 9, 2100 Copenhagen, Denmark; ^3^Department of Cardiology and Endocrinology, Hillerød University Hospital, Dyrehavevej 29, 3400 Hillerød, Denmark; ^4^Department of Integrated Healthcare, Bispebjerg University Hospital, Bispebjerg Bakke 11 B, 2400 Copenhagen, Denmark

## Abstract

The main aim of the study was to identify predictors of the effects of lifestyle intervention on diabetes mellitus type 2 patients by means of multivariate analysis. Data from a previously published randomised clinical trial, which compared the effects of a rehabilitation programme including standardised education and physical training sessions in the municipality's health care centre with the same duration of individual counseling in the diabetes outpatient clinic, were used. Data from 143 diabetes patients were analysed. The merged lifestyle intervention resulted in statistically significant improvements in patients' systolic blood pressure, waist circumference, exercise capacity, glycaemic control, and some aspects of general health-related quality of life. The linear multivariate regression models explained 45% to 80% of the variance in these improvements. The baseline outcomes in accordance to the logic of the regression to the mean phenomenon were the only statistically significant and robust predictors in all regression models. These results are important from a clinical point of view as they highlight the more urgent need for and better outcomes following lifestyle intervention for those patients who have worse general and disease-specific health.

## 1. Background

Diabetes mellitus type 2 (DM2) is a metabolic disorder that is characterised by high blood glucose in the context of insulin resistance and relative insulin deficiency. Severe complications can result from improperly managed DM2, including renal failure, blindness, slow healing of wounds, cardiovascular diseases, and erectile dysfunction. Changes in lifestyle, such as reduced physical activity, an unhealthy diet, and subsequent obesity, are the main causes of DM2. Systematic dietary treatment and/or exercise programmes have been shown to halve the risk of the development of DM2, as well as to lower blood sugar in diabetic patients [1].

The prevalence of DM2 is increasing in many countries including Denmark [2]. According to the Danish National Diabetes register, the number of patients diagnosed with diabetes (both types) in 2007 was 240,000, corresponding to about 4,000 diabetics per 100,000 Danes. In addition, many people with DM2 are thought to be undiagnosed [3].

The Integrated Rehabilitation for Chronic Conditions Project (SIKS) implemented lifestyle intervention programmes for people with chronic conditions, including DM2, within Copenhagen. The intervention was a rehabilitation programme including educational sessions or individual counselling, physical training, dietary instruction, and smoking cessation sessions. The development and implementation of the intervention took place from April 2005 to September 2007, was funded by the Ministry of Interior and Health, and involved cooperation between three organisations representing different sectors within the Danish health care system: (1) Bispebjerg Hospital (secondary health care; in general, responsibility for the administrative regions), (2) the municipality of Copenhagen (rehabilitation; in general, responsibility for the administrative municipalities), and (3) general practitioners (GPs) (primary health care and “gate keeping,” in general, private practice reimbursed by the administrative regions). The project was evaluated in terms of organisation, processes, and patient outcomes using both qualitative and quantitative measures [4, 5].

A detailed description of the evaluation of the intervention on DM2 patients can be found in Vadstrup, 2010 [6–8]. In brief, it was a randomised clinical trial comparing a rehabilitation programme, including standardised education and physical training sessions in the municipality's health care centre, and an equally long period of individual counseling in the diabetes outpatient clinic. The main finding was that the mean glycosylated hemoglobin (HbA1c) decreased significantly more among participants in the individual counseling group compared to the standardised rehabilitation programme. Both interventions similarly improved patients' weight, waist circumference, blood pressure, and some aspect of health-related quality of life.

## 2. Purpose

The main aim of this study was to explore which patient and/or treatment characteristics had an impact on the merged lifestyle intervention (i.e., in the municipality's health care centre and in the diabetes outpatient clinic) effect while distinguishing variables related to intervention organisation (i.e., health care centre versus diabetes outpatient clinic), diabetes care processes (i.e., urine, eye, and feet checks), and patients' health outcomes (i.e., results of general and disease-specific health and health-related quality of life assessments).

## 3. Methods

### 3.1. Assessments

A detailed description of the measures used to describe the patient sample before and after the intervention can be found in Vadstrup, 2010 [7].  Table 1 briefly presents the tests/questionnaires taken/responded to by DM2 patients to assess their health and quality of life; a more detailed description of some of the tests/questionnaires used follows.

**6-Minute Walk Test**

Patients above 60 years of age performed this test in an unobstructed indoor corridor. They walked 25 metres from one end of the corridor to the other at their own pace as many times as possible in 6 min. The total distance walked was measured in meters. The test is a feasible, reliable, and valid measure of functional capacity targeted at older people and people with moderate-to-severe physical impairment [9, 10]. (Patients under 60 years old underwent a cycling test, but the number of these patients was so small that they were not included in the analyses).

**SFT st and SFT 2.45**

During the SFT st, the patient had to sit on a regular chair without armrests and stand up as many times as possible in 30 seconds. The SFT 2.45 measured the time in seconds that a person required to rise from a regular chair without armrests, walk around a cone that was located 2.45 meters away, go back to the chair, and sit down again. Both tests have been proven to be valid and reliable in multiple studies [11].

**SF 36**

This is a valid and reliable questionnaire that has been used extensively in many languages. The SF 36 is a multipurpose health survey comprising 36 questions that measure eight domains: physical function, physical limitation, bodily pain, general health, vitality, social function, emotional limitation, and mental health. The first four domains can be merged into a summary Physical SF 36 (PSF 36), while the rest can be merged into the summary Mental SF 36 domain (MSF 36). The raw scores in each domain are transformed into 0 to 100 scales, with higher scores indicating superior quality of the relevant aspects of health-related quality of life. The scale has repeatedly shown high reliability and validity in multiple studies in many languages, including Danish [12].

**DSC-R**

This is a diabetes-specific questionnaire measuring the occurrence and perceived burden of diabetes-related symptoms in a broad comprehensive manner. It consists of 34 questions, which can be grouped into eight subscales. The total score ranges from 0 to 5 and is calculated by summing the item scores and dividing the sum by the number of items. A reduction in the score indicates an improvement in psychological and physiological distress. The DSC-R is known to be valid, reliable, and responsive to change [13].

### 3.2. Statistical Analyses

Uni- and bivariate analyses were used to describe the sample at baseline. Paired *t*-tests were used to identify the significant intervention-related changes or merged lifestyle intervention effects [14]. Multivariate linear regression models were run to explain the intervention effects, where statistically significant differences between pre- and postintervention outcomes were the dependent variables, and demographic characteristics, preintervention (i.e., baseline) outcomes, engagement with intervention, intervention-providing unit, and other intervention-related changes of statistical significance were entered as independent variables. To test the sensitivity of the results, analyses were conducted with complete data sets (i.e., replacing missing data with the means) [15]. SPSS 18.00 was used for all analyses.

## 4. Results

### 4.1. Sample

Measurements were taken from 143 diabetes patients at baseline (Table 2). Slightly more than half of the patients were males; the majority were of working age; 33.6% lived alone. The majority had a healthy lifestyle in terms of alcohol units consumed and time spent on exercise; 19.6% currently smoked. The results of the tests on exercise capacity were within the normal limits, with the exception of SFT 2.45, for which mean (SD) was 5.2 (4.42), the norm being 7–10 [11]. On average, patients were obese; mean BMI (SD) was 32.5 (6.27), the norm being up to 25 [16]. The BMI was significantly higher among women. On average, patients had mild hypertension; mean systolic/diastolic blood pressure (SD) was 147.7/84.7 (17.36/9.62), the norm being less than 130/80 mmHg [17]. Some of the disease-specific outcomes, such as glycaemic control (HbA1c treatment norm being up to 6.1% [17]), cholesterol (treatment norm being up to 4.5 mmol/L [17]), and LDL (treatment norm being up to 2.5 mmol/L [17]) were in the normal limits; however, HDL levels were lower (treatment norm being from 1.2 mmol/L for women, and from 1.7 mmol/L for men [17]) and triglycerides (treatment norm being up to 1.7 mmol/L [17]) were higher than the norm, indicating a slightly increased cardiovascular risk in the sample [18]. The scores on the general health-related quality of life questionnaire (SF 36) in the study population were close to those of healthy Danes aged 55–64 years [12]. The mean (SD) score on the diabetes-specific quality of life questionnaire (DSC-R) was 0.9 (0.64), which corresponded well with the mean score of this measure among diabetes patients with a BMI ranging from 30.0 to 39.9 [19]. The duration of diabetes in the sample was up to 10 years. The majority of patients were being monitored by their general practitioners (GPs) and more than half had had eye, feet, or urine checks during the past year.

The results of bivariate analyses of baseline outcomes were as follows: (a) older age correlated with decreased exercise capacity; (b) within the cardiovascular risk indicators, cholesterol was directly related to LDL, and triglycerides were inversely related to HDL; (c) BMI, blood pressure, HbAc1, and cardiovascular risk symptoms such as cholesterol, LDL, and triglycerides were mutually intercorrelated; (d) the outcomes from the different exercise capacity measures were mutually interrelated; (e) worse exercise capacity was associated with higher BMI and lower scores on SF 36—the emotional functioning subscale. In addition, patients who had had their eyes and feet checked during the past year had had diabetes for a longer time; and those who had had their feet checked during the past year had had significantly worse results in the exercise capacity tests as well as lower scores SF 36—the physical functioning and the bodily pain subscales.

### 4.2. Effects of the Intervention

Out of 143 patients, 53 (37.1%) completed the intervention programmes (i.e., complied with more than half of the prescribed rehabilitation activities).  Table 3 shows the statistically significant effects of the merged intervention calculated as the differences between post- and preintervention measurements.

In accordance with previously published reports [7], the intervention resulted in statistically significant improvements in DM2 patients' systolic blood pressure, waist circumference, exercise capacity, glycaemic control (HbA1c), and some aspects of general health-related quality of life.

### 4.3. Predictors of the Intervention Effects

Table 4 presents the results of the linear hierarchical regression models, in which the following independent variables were included: (1) sociodemographic characteristics: age and gender, (2) baseline outcomes: alcohol units, exercise hours, waist circumference, systolic blood pressure, one of the exercise capacity test results, SF 36—general health and SF 36—vitality scores for analogous SF 36 changes and MSF 36 for all other changes, logarithm (lg) to normalise distribution of disease duration, and eye-foot-urine checks, (3) unit in which the intervention took place, (4) completion of the intervention, and (5) other intervention-related changes of statistical significance forward inclusion. To better interpret the results, the outcome variables were standardised to give a positive value, that is, in cases in which the changes were negative (e.g., blood pressure had decreased), these changes were multiplied by (−1).

The regression model explained more than 60% of the change in systolic blood pressure. The baseline value (higher blood pressure at baseline-larger change) was the only significant and robust predictor in the model. Similar results were obtained from the model for the change in waist circumference. The models explained from 50% to almost 80% of the variance in the case of changes in exercise capacity, and the baseline values (worse exercise capacity at baseline-larger change) were significant and robust predictors in the models; additionally, disease duration negatively (longer disease-smaller change) and intervention unit (intervention in a health care centre, where exercise training was included in the programme-larger change) positively predicted the change in SFT st. The model for the change in glycaemic control (HbAc1) explained almost 60% of the variance, and the baseline value (higher HbAc1 at baseline-larger change) was the only significant and robust predictor. Similarly, the model for the change in cholesterol level predicted almost 70% of the variance, with the baseline value (higher cholesterol at baseline-larger change) being the only significant and robust predictor. The models for the changes in the two health-related quality of life dimensions explained 45% to 80% of the variances, with baseline values being significant and robust predictors in both models.

## 5. Discussion

The aim of this study was to determine which baseline health and health-related quality of life characteristics as well as diabetes care process-related variables (such as eye, foot, and urine checks), together with intervention-related variables (such as intervention-providing unit and engagement with intervention), predicted lifestyle intervention-related changes among DM2 patients. The results of multivariate linear regression models indicated that the relevant baseline outcomes were the largest and only statistically significant and robust predictors for all intervention effects, that is, pre- and postintervention changes in blood pressure, waist circumference, HbAc1, exercise capacity, and some aspects of general health-related quality of life.

The baseline characteristics of DM2 patients entering the intervention, the intervention effects and their predictors, and the validity of the study will be discussed in more detail below.

### 5.1. Study Population

As described in the Results section, the study population comprised relatively healthy middle-aged Danes, who had been diagnosed with DM2 and on average were slightly obese and had mild hypertension as well as mildly increased cardiovascular risk. In terms of the general functioning and quality of life outcomes, the only difference from healthy Danes of similar age was a slightly lower exercise capacity, which was anticipated given that a decrease in exercise capacity is usually related to increased body mass [20, 21]. Disease-specific functioning and quality of life outcomes corresponded well with analogous outcomes among DM2 patients with a similar body mass [19]. The results of bivariate analyses of baseline outcomes (i.e., reduced exercise capacity among older patients; mutual association between glycaemic control, BMI, and cardiovascular risk indicators, such as blood pressure and lipid profile; correlation between exercise capacity and mental quality of life component) were consistent with findings/conventions reported in the literature [21–28]. Additionally, the patients, who had had their eyes and feet checked during the past year, had been diagnosed with DM2 for longer and were less physically fit, and had lower scores on the quality of life survey's subscales addressing issues related to physical health. We suggest that the more frequent eye and feet checks in the study sample indicated more severe disease rather than better quality of diabetes care process. According to the literature, patients participating in rehabilitation activities are more motivated to change their health status [29]. Thus, all patients who volunteered to participate in the life-style intervention analysed most probably took good care of themselves, and therefore those who were/felt more ill came to eye and feet checks more frequently comparing to those who were/felt less ill.

### 5.2. Intervention Effects and Their Predictors

The effects of lifestyle interventions on DM2 patients are relatively well described in the literature and the effects seen in this study corresponded well with those described previously [30–33]. However, few studies have attempted to explain these effects using multivariate analysis. We screened the PubMed abstracts of the past 10 years, which we retrieved using the following combination of keywords: diabetes mellitus type 2, rehabilitation/exercising/education, and multivariate analysis/regression, and we identified only a few studies [34–38]. The vast majority of these studies focused on the multivariate explanation of the effect of exercise training on glycaemic control, and one of them looked at the effect of training on cardiovascular risk indicators [37].

Thus, our study contributes to the literature by presenting multivariate analyses of the effects of more comprehensive lifestyle intervention on DM2 patients in general and on exercise capacity and quality of life in particular. The latter will be touched upon later. With regard to exercise capacity, it was shown that changes in exercise capacity were dependent on the duration of disease, which, in turn, might have been related to age. Study participants who had had DM2 for a longer period (and who were probably older) showed less improvement in their exercise capacity than those patients who had been diagnosed with DM2 recently (and who were probably younger). Older age was previously reported to be related to a lower exercise capacity in DM2 patients [21]. Our results additionally showed that older patients are not only physically weaker but also exhibit smaller improvements in their physical fitness during lifestyle interventions. Changes in exercise capacity were also predicted by the unit, in which the intervention took place. Changes were greater among those patients who attended a health care centre for the intervention, where, unlike the individually counselled patients, they also participated in a physical training programme. Thus, physical training certainly has an impact on changes in exercise capacity among DM2 patients as well as other patients with chronic condition [39].

With regard to the change in glycaemic control and cardiovascular risk (i.e., blood pressure and lipid profile), our data confirmed previous findings. For example, the results of our study, as well as those of previously conducted studies, showed that the change in HbA1c was dependent on whether the patients completed all the rehabilitation activities including exercise training or not [34, 35], and that the larger changes in HbA1c were related to higher levels of HbAc1 at the start of the intervention [38].

In conclusion, our study clearly shows that when predicting the effects of lifestyle intervention on DM2 patients by means of multivariate linear regression models, the largest in size and only statistically significant and robust predictors of all intervention-related changes are variables evidencing what is generally known as “regression to the mean” phenomenon. In statistics, “regression to the mean” is the phenomenon that if a variable is extreme on its first measurement, it will tend to be closer to the average on a second measurement, and if it is extreme on a second measurement, it will tend to have been closer to the average on the first measurement. In our study, that is, the poorer selected variables at baseline, the larger the improvement of these outcomes following the rehabilitation. Such a result, though had been expected statistically, is still important from a clinical point of view. It highlights the urgent need for and better outcome of lifestyle interventions for those DM2 patients who are physically weak, have poor glycaemic control and high cardiovascular risk, and assess their general health-related quality of life as being poorer. Regarding the latter, the results showed that only a couple of SF 36 survey subscales, namely, general health and vitality, improved significantly after the intervention. It is relatively common that DM2 patients' general health-related quality of life is fairly similar to the one among healthy people of a similar age, which was also the case in our study [40, 41]. Therefore, following the previously outlined logic of regression to the mean, small and not significant changes in the rehabilitated patients' SF 36 outcomes could have been anticipated.

### 5.3. Validity of the Study

The arguments supporting validity of the study were that only well-known, valid, reliable, and feasible measures were used to assess patient outcomes before and after the intervention. Moreover, some constructs, such as exercise capacity, were assessed by several tests. The fact that the descriptive characteristics of the study population were similar to those of other populations described previously confirms the validity of the descriptive results. The limitations, however, include a possible selection bias as the patients entered the intervention voluntarily. Moreover, due to the absence of standardised recording and the relatively small sample size, we did not account for other important predictors which could influence the results, for example, comorbidities as well as antidiabetic, antihypertensive, and lipid-lowering therapy changes during the study [42].

Finally, the study was not specifically designed to examine the investigated problems; we used previously collected data to determine whether the effect of a newly introduced rehabilitation programme in the municipality's health care centre would differ from that of individual counselling carried out for the same length of time in the diabetes out-patient clinic. As the effects of the interventions performed in these two health care facilities did not differ to any great extend, the data were merged to increase the sample size and thus to improve the statistical power of the multivariable analyses.

## Figures and Tables

**Table 1:** Pre- and postintervention measurements among DM2 patients.

Subject of assessment	Tests and instruments used for assessment
General health	Weight, body mass index (BMI), waist circumference, systolic blood pressure (BP systolic), and diastolic blood pressure (BP diastolic)
General functioning	6-minute walk test, stand and sit standard senior fitness test (SFT st), and 2.45-minute up and go senior fitness test (SFT 2.45)
Disease-specific functioning	Glycosylated hemoglobin (HbA1c), cholesterol, high-density lipoproteins (HDL), low-density lipoproteins (LDL), and triglycerides
General and disease-specific health-related quality of life	Short form 36 (SF 36), diabetes symptom checklist-revised (DSC-R)

**Table 2:** Sample characteristics before intervention (*N* = 143).

Group of variables	Variable	Outcomes in means (SD)*or %
	Gender	
	Men	59.4
	Women	40.6
	Age	58.2 (9.65)
	Ethnicity	
	Danes	90.9
	Other nationalities	9.1
	Civil status	
Sociodemography	With partner	66.4
	Alone	33.6
	Job situation	
	In job	39.9
	Out of job	7.0
	Retired	53.1
	Education	
	High school/special technical	44.8
	Grammar school/university	55.2

	Alcohol units per week	Median 2.0 (IQR 1.0–7.0)
	Smoking status	
	Smokers	19.6
Lifestyle	Never smokers	36.4
	Quit smokers	44.0
		Median 6.5
	Exercising hours per week	(IQR 4.0–11.8)

	BMI	32.5 (6.27)
General health	Waist circumference	108.8 (14.90)
	Blood pressure systolic/diastolic	147.7/84.7 (17.36/9.62)

	6-minute walk test in meters (*N* = 68)	524.8 (112.54)
General functioning	SFT st in times per 30 seconds	16.1 (4.28)
	SFT 2.45 in seconds	5.2 (1.42)

	HbA1c in %	7.9 (0.84)
	Cholesterol in mmol/L	4.8 (1.05)
Disease-specific functioning	LDL in mmol/L	2.7 (0.95)
	HDL in mmol/L	1.2 (0.34)
	Triglycerides in mmol/L	2.3 (1.52)

	SF 36—physical functioning	80.6 (19.98)
	SF 36—physical role functioning	72.5 (35.82)
	SF 36—bodily pain	80.0 (23.78)
	SF 36—general health perception	64.0 (19.67)
	SF 36—vitality	60.0 (23.64)
General and disease-specific health-related quality of life	SF 36—social role functioning	87.5 (21.13)
	SF 36—emotional role functioning	75.90 (35.85)
	SF 36—mental health	78.0 (18.70)
	SF 36—physical component merged	74.9 (19.76)
	SF 36—mental component merged	75.1 (20.72)
	DSC-R	1.0 (0.62)

	Health care professional taking care of diabetes	
	General practitioner	74.8
	Endocrinologist	25.2
History of the disease and its treatment	Length of diabetes in years	6.6 (6.43)
	Having eye check in the past year	64.3
	Having foot check in the past year	63.6
	Having urine check in the past year	67.8

Abbreviations: *N*: sample size; SD: standard deviation, unless otherwise indicated; IQR: interquartile range for not normally distributed outcomes.

**Table 3:** Statistically significant effects of the merged lifestyle intervention.

Group of variables	Variable	Pre-post difference mean [SE] (*N*)
General health	Waist circumference in cm	−3.8 [1.96]* (121)
	Blood pressure systolic in mm Hg	−5.2 [1.27]* (121)
General functioning	SFT st in times per 30 seconds	2.05 [0.65]* (95)
	SFT 2.45 in seconds	−0.4 [0.19]* (95)
Disease-specific functioning	HbAc1 in %	−0.5 [10.12]** (117)
	Cholesterol in mmol/L	−0.3 [0.13]* (121)
General and disease-specific health-related quality of life	SF 36—general health perception	4.3 [1.97]* (97)
	SF 36—vitality	6.3 [2.80]* (95)

*P < 0.05; **P < 0.001, SE: standard error of the mean, *N*: sample size.

**Table 4:** Parameters of the multivariable linear regression models to explain intervention effects.

Group of variables	Dependent variables	Adjusted* R *square % (*N*)	Statistically significant predictors: standardized coefficient beta
General health	Blood pressure systolic change (multiplied by (−1))	61.4** (61)	Blood pressure systolic baseline: 0.754** Exercising baseline°: 0.266*
	Waist circumference change (multiplied by (−1))	61.2** (66)	Waist circumference baseline: 0.745** Blood pressure systolic change°: 0.420*

General functioning	SFT st change	51.3* (65)	SFT st baseline: −0.607** Lg disease length: −0.296* Unit*¹*: 0.349*
	SFT 2.45 change (multiplied by (−1))	77.3* (61)	SFT 2.45 baseline: 0.777** Blood pressure systolic baseline°: 0.285*

Disease-specific functioning	HbAc1 change (multiplied by (−1))	59.7* (61)	HgbAc1 baseline: 0.610** Completion of intervention² °: 0.181* BP systolic change (multiplied by (−1))°: 0.377* Cholesterol change (multiplied by (−1))°: 0.459*
	Cholesterol change (multiplied by (−1))	67.6* (61)	Cholesterol baseline: 0.925** Lg disease length°: 0.198*

General health-related quality of life	SF 36 general health change	45.5** (64)	SF 36—general health baseline: −0.731** Alcohol units baseline°: −0.293* Waist circumference baseline°: 0.366*
	SF 36 vitality change	79.8** (65)	SF 36—vitality baseline: −0.754**

*¹*: reference group: outpatient clinic; ²: reference group: those who did not complete the intervention; *: *P* < 0.05; **: *P* < 0.001, °: sensitive to missing data imputation.
